# Validation of the Arabic Version of the Long-Term Conditions Questionnaire (LTCQ): A Study of Factor and Rasch Analyses

**DOI:** 10.3390/healthcare13131485

**Published:** 2025-06-20

**Authors:** Walid Al-Qerem, Salwa Abdo, Anan Jarab, Alaa Hammad, Judith Eberhardt, Fawaz Al-Asmari, Lujain Al-Sa’di, Razan Al-Shehadeh, Dana Khasim, Ruba Zumot, Sarah Khalil, Ghazal Aloshebe, Jude Aljazazi

**Affiliations:** 1 Department of Pharmacy, Faculty of Pharmacy, Al-Zaytoonah University of Jordan, Amman 11733, Jordan; waleed.qirim@zuj.edu.jo (W.A.-Q.); phres01@zuj.edu.jo (L.A.-S.); danaeyhab99@gmail.com (D.K.); ghazalakram19@gmail.com (G.A.);; 2 Department of Clinical Pharmacy, Faculty of Pharmacy, Jordan University of Science and Technology, P.O. Box 3030, Irbid 22110, Jordan; anan.jarab@aau.ac.ae; 3 Department of Psychology, School of Social Sciences, Humanities and Law, Teesside University, Middlesbrough TS1 3BX, UK; j.eberhardt@tees.ac.uk; 4 Department of Pharmacology and Toxicology, College of Pharmacy, King Saud University, Riyadh 12372, Saudi Arabia; ffalasmari@ksu.edu.sa

**Keywords:** LTCQ, chronic conditions, EQ-5D, quality of life, PROMs

## Abstract

**Background:** Patient-reported outcome measures (PROMs) are essential for capturing the lived experiences of individuals managing chronic diseases. However, few PROMs have been culturally adapted and validated for Arabic-speaking populations. **Aim:** This study aimed to translate, culturally adapt, and validate the Long-Term Conditions Questionnaire (LTCQ) for use among Arabic-speaking adults living with chronic diseases in Jordan. **Methods:** Following forward–backward translation and an expert review, a cross-sectional survey of 1057 adults with chronic illnesses was conducted. The psychometric evaluation involved exploratory and confirmatory factor analyses (EFA and CFA) and Rasch modelling. While the original LTCQ assumed a unidimensional structure, EFA and CFA supported a two-factor solution: Empowerment and Functional Wellbeing, and Health-Related Psychosocial Distress. **Results:** The Rasch analysis confirmed that the item response thresholds were ordered, with good item targeting, and no differential item functioning (DIF) by gender. The removal of one poorly performing item resulted in a refined 19-item scale with strong reliability and validity. **Conclusions:** The Arabic LTCQ demonstrated robust psychometric properties and cultural relevance, supporting its use in clinical care, research, and policy initiatives. Future work should examine longitudinal responsiveness and further validate the tool across diverse Arabic-speaking populations.

## 1. Introduction

Non-communicable chronic diseases (NCCDs), defined by irreversible pathological changes affecting various body systems, have become increasingly prevalent across the globe [1]. In recent years, the incidence of chronic diseases has risen markedly, with clear consequences for patients’ quality of life [2,3]. Conditions such as diabetes, stroke, cancer, cardiovascular diseases, arthritis, and respiratory disorders have long been leading causes of mortality and disability in developed nations. Globally, long-term conditions now account for over 70% of all deaths [4]. Health care systems face mounting challenges in this context, particularly with the growing proportion of elderly individuals, who are at higher risk for developing chronic diseases and often struggle to maintain a good quality of life as they age [5]. Yet, “living well” with a chronic condition is no simple matter; it touches upon diverse aspects of health-related quality of life, including the symptom burden, mobility, social engagement, emotional wellbeing, and, as increasingly recognized, domains such as self-efficacy, the care burden, which refers to the practical, emotional, and social demands placed on individuals and their families as they manage daily treatment tasks, appointments, and disease-related challenges, and confidence in managing one’s own health [6].

In many low- and middle-income countries, the future trajectory of health care needs will be shaped predominantly by non-communicable diseases and driven by a confluence of factors: declining rates of communicable diseases, improvements in maternal and child health, lifestyle shifts such as increased smoking rates, and ongoing demographic aging [7]. Jordan, like many countries in the region, faces a similar pattern. Non-communicable diseases are now the primary drivers of morbidity and mortality. Approximately 5.6% of Jordanians are aged 60 years or older, and the population is increasingly exposed to major risk factors such as tobacco use, sedentary behaviour, and obesity [8,9]. Older adults, in particular, often require more specialized and continuous medical care given their higher burden of chronic illnesses.

Patient-reported outcome measures (PROMs) have become essential tools for capturing patients’ perspectives on their health status, quality of life, and daily functioning, offering insights that are often invisible through clinical measures alone. However, selecting appropriate and validated instruments remains challenging, especially amid the expanding literature in this field [10,11]. Among the available measures, the Long-Term Conditions Questionnaire (LTCQ) stands out due to several unique features. Unlike generic PROMs that may focus narrowly on physical symptoms or single-disease contexts, the LTCQ was specifically designed to capture the broader concept of “living well” with one or more chronic conditions. Its development process involved not only rigorous research synthesis but also extensive consultations with patients, healthcare professionals, and methodologists, ensuring that the tool reflects both lived experiences and clinical priorities. It balances the assessment of both functional wellbeing and psychosocial dimensions, which are often underrepresented in other PROMs. Furthermore, it is intended for use across a wide range of chronic diseases and care settings, making it especially beneficial for real-world settings where multimorbidity and complex health needs are common. These attributes make the LTCQ a particularly robust and patient-centred instrument for evaluating the quality of life of individuals with long-term conditions [12].

### Objectives

Although several PROMs exist, few address the experience of managing multiple chronic illnesses comprehensively, and even fewer have been adapted for Arabic-speaking populations. This study addresses this gap by aiming to translate and validate the LTCQ into Arabic. To date, no Arabic version exists, despite the pressing need for tools that reflect the lived experiences of patients managing complex health conditions in Jordan and across the Middle East. By adapting the LTCQ, a measure that captures both functional wellbeing and psychosocial aspects of living with chronic diseases, this study seeks to provide a culturally and linguistically appropriate instrument to support patient-centred care and health system improvements in Arabic-speaking contexts.

## 2. Materials and Methods

In this cross-sectional study, participants were recruited through a multi-modal community-based approach designed to reach a broad spectrum of adults living with chronic conditions in Jordan. An online questionnaire was developed using Google Forms. The questionnaire link, consent form, and a summary of the study objectives were distributed through community centres, pharmacies, outpatient clinics, and various Jordanian social media platforms. Participants were asked to fill out the consent form before proceeding to the questionnaire. This strategy ensured accessibility to individuals actively engaged in their own healthcare but not currently hospitalized or receiving inpatient care. Data collection was intentionally not conducted within hospital wards, as the study sought to capture experiences from stable, community-dwelling adults in typical outpatient or daily living environments.

Responses were collected from September 2024 to April 2025. Inclusion criteria included being a Jordanian resident aged 18 or older living with one or more chronic physical, mental, or neurological health conditions. To confirm that participants met the inclusion criteria, demographic questions regarding age, place of residency, nationality, and chronic conditions were incorporated at the beginning of the questionnaire.

The authors obtained ethical approval from the Institutional Review Board and the Deanship of Research at Al-Zaytoonah University of Jordan. This study followed the Declaration of Helsinki’s ethical guidelines. Ethical approval was granted on 10 September 2024 (2025-2024/07/42).

### 2.1. Data Collection and Study Instruments

The questionnaire contained three sections. The first section was a demographic data collection sheet, and the second included the Long-Term Conditions Questionnaire (LTCQ) [6]. The questionnaire consists of 20 Likert-scale items, with response options ranging from 0 (“Never”) to 4 (“Always”); items 9–15 were negatively phrased and were therefore reverse-scored. A sum of the 20 item scores was calculated and rescaled to produce an overall LTCQ score ranging from 0 to 100, with higher scores indicating a better level of “living well” with long-term conditions [6]. Moreover, the EuroQol five-dimension, three-level version questionnaire (EQ-5D) was included in the third section to assess the predictivity of the study’s questionnaire [13]. It is a standardized and well-validated tool designed to measure the health-related quality of life of people living with chronic conditions. This instrument focuses on five dimensions of patients’ health, including mobility, self-care, usual activities, pain/discomfort, and anxiety/depression, with three response levels for each dimension (no problems, some problems, and extreme problems).

### 2.2. Tool Validation

The content validity of the questionnaire was evaluated by a group of experts comprising a cardiologist, an endocrinologist, an internist, and two clinical pharmacists. To accommodate the study sample’s native language, Arabic, the questionnaire was translated using a forward–backward translation process conducted by different translators. A pilot study was conducted with 25 Jordanian individuals living with chronic diseases to assess the questionnaire’s suitability and clarity; data from this pilot phase were excluded from the final statistical analysis. Additionally, several statistical methods were applied to ensure comprehensive validation of the tool. These methods included computing Cronbach’s alpha, conducting a factor analysis, and performing a Rasch analysis.

### 2.3. Sample Size Calculations

The item–subject ratio approach was applied in order to determine the minimum sample size; widely cited item–subject ratios range from 5 to 20 [14,15,16]. To ensure this study’s reliability, validity, and generalizability, the highest suggested ratio of 1:20 was applied. Given that the LTCQ included 20 items, the minimum required sample size was 400. However, the present study included a substantially larger sample of 1052 participants to further increase the reliability, generalizability, and validity of the results.

### 2.4. Statistical Analysis

The original one-factor model suggested by the original questionnaire was assessed using a confirmatory factor analysis (CFA); however, the model fit was inadequate. Therefore, to evaluate the latent structure of the 20-item questionnaire in the present dataset, the sample was randomly split into two equal subsamples for cross-validation. The first subsample (n = 528) was subjected to an exploratory factor analysis (EFA), while the second subsample (n = 529) was used for the CFA. All items were measured on a 5-point Likert-type ordinal scale ranging from 0 to 4. Given the ordinal nature of the data, polychoric correlations were computed, and the EFA was conducted using the minimum residual (MinRes, Osborne Park, Australia) extraction method with oblimin rotation in the psych package (version 2.5.3). The number of factors to retain was guided by a parallel analysis and the scree plot. The Kaiser–Meyer–Olkin (KMO) Measure of Sampling Adequacy and Bartlett’s Test of Sphericity were conducted to assess the suitability of the data for the factor analysis. Communalities and factor loadings were assessed; items with low communalities, low factor loadings, or substantial cross-loadings were excluded from the analysis.

Subsequently, the CFA was used to assess the final model produced by the EFA results using the other half of the dataset. The analysis was conducted using the lavaan package (version 0.6-17) in R, employing the WLSMV estimator (Weighted Least Squares Mean and Variance adjusted), which is appropriate for categorical ordinal data. Model fit was assessed using multiple indices, including χ^2^/df (minimum discrepancy), the Comparative Fit Index (CFI), the Standardized Root Mean Square Residual (SRMR), the Root Mean Square Error of Approximation (RMSEA), and the Tucker–Lewis Index (TLI). For the model fit evaluation, the following criteria were applied: a χ^2^/df ratio less than 5 was considered acceptable [17]; RMSEA and SRMR values ≤0.08 indicated an acceptable fit; for the TLI, a value of 1 represented a perfect fit, while values approaching 1 suggested a very good fit [14]; and the CFI ranged from 0 to 1, with 1 indicating a perfect fit and values ≥0.9 reflecting an excellent fit [18]. Furthermore, factor loadings produced by the CFA were also assessed. Cronbach’s alpha was computed for each scale, with an acceptable value of ≥0.7 [19].

The Rasch analysis was conducted to reconfirm the suitability of the proposed model structure and to evaluate the hierarchy of the model items. Furthermore, the Rasch analysis was used to assess the ability of the questionnaire to differentiate between participants’ quality of life levels.

Person reliability and item separation reliability were computed to verify the suitability of the model. Additionally, infit and outfit statistics were produced. Items’ infit and outfit mean square (MSE) values ranging between 0.5 and 1.5 were considered acceptable [20]. Items’ Thurstonian thresholds were computed, and the category order of each item was assessed. A Differential Item Functioning (DIF) analysis between genders was conducted, with a DIF value of <0.43 logits considered acceptable [20]. To visually investigate item locations, thresholds, and participants’ distribution, a Wright map was generated. Moreover, the predictivity of the questionnaire was assessed by validating it against the EuroQol 5 Dimensions (EQ-5D) questionnaire [21].

## 3. Results

The sociodemographic profiles of the participants are presented in Table 1. A total of 1057 chronic disease patients were included in this study; most were female (63.8%), with a median age of 42 years (interquartile range: 28–55 years). Most participants were married (62.6%) and held a university or college degree (66.6%). Furthermore, more than half of the participants earned a monthly income of less than 500 JOD. About 76.4% of participants had only one chronic disease, and the median disease duration was 5 years (interquartile range: 1–10). Finally, approximately 26.9% of participants had public medical insurance.

The LCTQ item are distributed between “positive” and “negative” items; for the positive statements, the most frequently selected option for most items was “Often.” The item with the highest percentage of “Often” responses was “Felt in control of daily life” (38.3%). In contrast, among the reverse-coded statements, “Never” was the most commonly selected option for most items (Table 2).

When conducting the CFA on the initial one-factor model, the results demonstrated a poor fit: CFI = 0.74, TLI = 0.71, RMSEA = 0.073, and SRMR = 0.128. These values are lower than the generally accepted thresholds for a good model fit (CFI and TLI values ≥0.9, and RMSEA and SRMR values ≤0.08). Additionally, LTCQ 9 exhibited an unacceptably low factor loading (0.147), further suggesting a poor model fit. Given these results, an exploratory factor analysis (EFA) was conducted to identify a more appropriate structure. The Kaiser–Meyer–Olkin (KMO) measure and Bartlett’s test of sphericity confirmed the suitability of the data for the EFA (KMO = 0.91, *p* < 0.001). The parallel analysis and the scree plot supported a two-factor solution, Empowerment and Functional Wellbeing and Health-Related Psychosocial Distress, with the first two eigenvalues (6.16 and 3.38) exceeding the simulated random values.

LTCQ 9 demonstrated consistent issues across both the EFA and CFA. In the EFA, it exhibited low communality (h^2^ = 0.30), with a primary loading of 0.59 on Factor 2 and a notable cross-loading (−0.24) on Factor 1, indicating poor alignment with a single construct. The CFA further confirmed its problematic nature, showing a weak standardized loading (0.333, below the 0.50 threshold) and a high modification index (MI = 119.30) for cross-loading onto Factor 1. These findings suggested that LTCQ 9 contributed significantly to model misfit, violated simple structure assumptions, and increased residual variance. Following its removal, the model was re-estimated with the remaining 19 items. This resulted in a substantially improved model. For example, communalities in the 19-item model ranged from 0.31 to 0.65, and the standardized factor loadings produced by the EFA ranged from 0.55 to 0.77 (Table 3).

The median score for the Empowerment and Functional Wellbeing factor (Factor 1) was 63.46 (interquartile range: 50–76.92) out of a maximum possible score of 100, with a Cronbach’s alpha of 0.90. For the Health-Related Psychosocial Distress factor, the median score was 62.85 (interquartile range: 50–83.35) out of 100, with a Cronbach’s alpha of 0.83.

The final two-factor CFA solution, applied using the second half of the data, demonstrated a good model fit, with a CFI = 0.945, TLI = 0.936, RMSEA = 0.067 (90% CI: 0.060–0.074), SRMR = 0.057, and χ^2^/df = 4.6. The standardized factor loadings ranged from 0.48 to 0.86 (Table 2).

### Rasch Model Analysis

Using the complete dataset, a Rasch analysis was applied to the suggested two-factor, 19-item model. The results indicated that the item separation reliability and person reliability for the Empowerment and Functional Wellbeing and Health-Related Psychosocial Distress factors were 0.90, 0.83, 0.881, and 0.748, respectively. Infit and outfit values confirmed the tool’s ability to differentiate between participants, and all values fell within acceptable ranges (Table 3).

The thresholds displayed in Table 3 indicated that all items had ordered response categories. The distribution of item thresholds across various difficulty levels revealed differing levels of challenge for participants; the easiest threshold was the first threshold of LTCQ 10, followed by LTCQ 15, while the most challenging thresholds were the fourth threshold of LTCQ 16. The item difficulty analysis indicated that LTCQ 1 (location = −0.323) and LTCQ 8 (location = −0.328) were the most difficult items, whereas LTCQ 7 (location = −1.189) was the least difficult.

**Figure 1:**
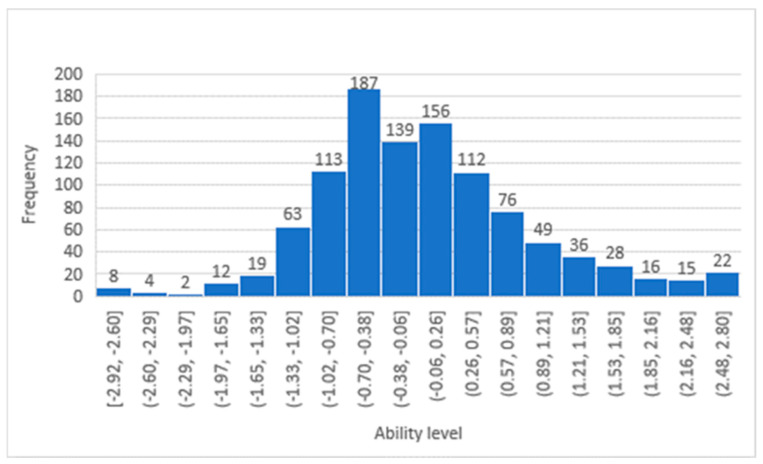
Participants’ ability distribution in the Empowerment and Functional Wellbeing factor.

**Figure 2:**
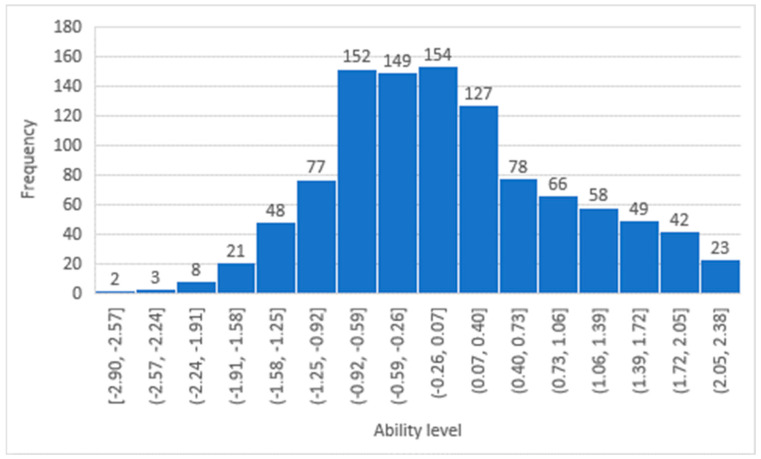
Participants’ ability distribution in the Health-Related Psychosocial Distress factor.

The participants’ ability histograms, shown in Figure 1 and Figure 2, confirm that the patients’ responses were distributed across all levels of difficulty, and the largest portion of the sample was situated in the middle range. The DIF between the two genders was evaluated, as shown in Table 3. There were no significant differences between males and females across all 19 items. While Modern Standard Arabic is widely understood. When validating the studied questionnaire (LTCQ) against the well validated EQ-5D questionnaire, the person correlation coefficient was 0.4, indicating a reasonable correlation.

## 4. Discussion

This study translated, culturally adapted, and validated the LTCQ for Arabic-speaking adults with chronic illnesses in Jordan. The Arabic version showed strong psychometric performance, supporting its use as a patient-centred tool to assess quality of life in this population.

Consistent with the original English LTCQ validation, our findings confirmed the instrument’s ability to capture both the functional and psychosocial dimensions of chronic diseases [6]. However, whereas the original study supported a unidimensional structure, our data revealed a two-factor solution: Empowerment and Functional Wellbeing, and Health-Related Psychosocial Distress. This suggests that Arabic-speaking individuals may conceptualise chronic illness along two distinct but related axes—one centred on self-management and daily functioning, and the other on psychological and social burdens. This distinction may reflect cultural norms, including strong familial support and resilience on the one hand, and the stigma surrounding illness on the other [6]. Such patterns are consistent with findings from cultural psychology, where collectivist societies, such as many in the Arab world, are characterised by interdependence, communal coping, and social responsibility [22,23]. However, illness and mental health may be stigmatised due to concerns about honour and social judgment [24], which could explain the emergence of psychosocial distress as a distinct latent factor.

These findings reinforce the importance of culturally adapting PROMs. Measures developed in one context cannot be assumed to function similarly in others; local validation ensures conceptual relevance and measurement accuracy [22].

The Rasch analysis further confirmed the Arabic LTCQ’s reliability. All items showed ordered thresholds, appropriate targeting, and good item/person separation [25]. The Wright map demonstrated that the item difficulty matched the distribution of respondents’ abilities, with no ceiling or floor effects. The DIF analysis indicated no gender bias, ensuring fairness in responses across groups [26]. Item 9 (“Felt bothered by symptoms”) showed poor performance and was removed, yielding a refined 19-item tool with improved model fit [22].

The Arabic LTCQ offers a practical tool for clinicians, researchers, and policymakers working to improve outcomes for Arabic-speaking populations with long-term conditions. Its comprehensive scope supports the growing emphasis on holistic, patient-centred models of care [10,12].

Its moderate correlation with the EQ-5D reflects differences in focus: while the EQ-5D emphasises physical health, the LTCQ also captures social and emotional dimensions, suggesting that the two tools are complementary rather than interchangeable.

In clinical practice, PROMs like the LTCQ help identify not just physical symptoms but also broader psychosocial needs. Their use has been linked to better communication, greater patient satisfaction, and more tailored care [27,28]. In research, the LTCQ offers a validated outcome for studies targeting self-management and quality of life [29]. At the policy level, PROMs inform value-based healthcare, guide service improvement, and enhance system responsiveness to patient experiences [30,31]. Given the rising non-communicable disease burdens in Arabic-speaking countries, locally adapted tools like the LTCQ are essential for effective, culturally relevant care [32].

### Strengths, Limitations, and Future Directions

This study employed a rigorous multi-method validation process, including both Classical Test Theory and Rasch modelling, and drew on a large, diverse sample, enhancing generalisability. Translation followed established best practices and was further refined through expert review and piloting.

However, participants were recruited solely from Jordan. While Modern Standard Arabic is widely understood, future studies should assess the LTCQ’s performance across other Arabic-speaking countries and clinical subgroups, particularly in North African countries like Morocco and Mauritania. The cross-sectional design also prevented an examination of test–retest reliability or responsiveness over time. These areas should be prioritised in future research [22]. Although gender-based DIF was not observed, other sociodemographic variables warrant investigation. Furthermore, the study sample overrepresented individuals working in the medical field, who comprised approximately 23% of the sample. While this sample distribution does not mimic the studied population, it provides an opportunity to examine the unique challenges and quality of life issues encountered by healthcare professionals living with chronic conditions. The inclusion of this subgroup offers valuable insights into how medical expertise may shape disease management behaviours and health-related quality of life. Moreover, the large overall sample enabled subgroup analyses, supporting a detailed understanding of quality-of-life differences among various occupational groups. Another limitation of this study is that it did not collect specific information on the clinical severity of participants’ chronic conditions. Our data collection relied on self-reported diagnoses and did not include clinical validation or stratification by disease severity. Additionally, because the study was conducted among community-dwelling and outpatient individuals rather than hospitalized patients, our sample likely underrepresents those with the most severe or acute disease states. Therefore, the findings should be interpreted with this context in mind.

Further validation across regions, integration into electronic health records, and longitudinal studies assessing sensitivity to clinical change are recommended. Embedding the LTCQ into EHRs could enhance the routine monitoring of patient-reported outcomes, facilitate personalised care planning, and support data-driven decision-making. For example, the integration of PROMs such as the EQ-5D into NHS digital systems has improved the systematic collection of patient experience data and informed service evaluations [27,30]. Furthermore, qualitative work could enhance cultural nuances and improve item clarity or relevance.

## 5. Conclusions

The Arabic LTCQ is a culturally appropriate, psychometrically robust tool for assessing the lived experiences of Arabic-speaking individuals with chronic illnesses. The final validated measure captures two key dimensions of living with chronic disease—Empowerment and Functional Wellbeing, and Health-Related Psychosocial Distress—offering a comprehensive assessment of quality of life. These distinct but complementary factors enhance the tool’s clinical utility and cultural relevance. Its strong measurement properties support its use in clinical, research, and policy contexts. Future research should extend its validation to other settings, assess longitudinal responsiveness, and explore its integration into patient-centred care models across the Arabic-speaking world.

## Figures and Tables

**Table 1:** Sociodemographic profiles of the study participants.

		Count (%) or Median (IQR)
Age		42 (28–55)
Gender	Female	674 (63.8%)
	Male	383 (36.2%)
Education	High school or less	352 (33.4%)
	College/University degree	702 (66.6%)
Average monthly income (Jordanian Dinar) *	<500	577 (54.6%)
	500–1000	348 (32.9%)
	>1000	132 (12.5%)
Marital status	Not married	395 (37.4%)
	Married	662 (62.6%)
Health insurance	Public	284 (26.9%)
	Private	209 (19.8%)
	Military	136 (12.9%)
	Not insured	428 (40.5%)
Number of medications used		2 (1–4)
Disease duration (years)		5 (1–10)
Number of chronic diseases		1 (76.4%)
		2 (16.2%)
		≥3 (7.4%)
Working in the medical field	No	768 (72.7%)
	Yes	289 (27.3%)

* Note: 1 Jordanian Dinar equals approximately 1.40 US dollars.

**Table 2:** Participants’ responses to LTCQ items.

	Never	Rarely	Sometimes	Often	Always
	Count (%)				
1. Able to cope well with health conditions	99 (9.4%)	107 (10.1%)	342 (32.4%)	338 (32%)	171 (16.2%)
2. Able to fulfil responsibilities	57 (5.4%)	94 (8.9%)	251 (23.7%)	386 (36.5%)	269 (25.4%)
3. Able to be as physically active as you wanted	59 (5.6%)	134 (12.7%)	374 (35.4%)	335 (31.7%)	155 (14.7%)
4. Felt in control of daily life	55 (5.2%)	105 (9.9%)	286 (27.1%)	405 (38.3%)	206 (19.5%)
5. Able to take part in activities you enjoy	62 (5.9%)	144 (13.6%)	324 (30.7%)	358 (33.9%)	169 (16%)
6. Felt that your home is suitable for your needs	70 (6.6%)	104 (9.8%)	219 (20.7%)	347 (32.8%)	317 (30%)
7. Felt safe at home	41 (3.9%)	75 (7.1%)	160 (15.1%)	261 (24.7%)	520 (49.2%)
8. Felt safe outside the home	95 (9%)	147 (13.9%)	320 (30.3%)	317 (30%)	178 (16.8%)
9. Felt bothered by symptoms *	142 (13.4%)	128 (12.1%)	363 (34.3%)	249 (23.6%)	175 (16.6%)
10. Felt more dependent on others than you wanted *	280 (26.5%)	311 (29.4%)	267 (25.3%)	144 (13.6%)	55 (5.2%)
11. Felt lonely due to health conditions *	369 (34.9%)	216 (20.4%)	255 (24.1%)	154 (14.6%)	63 (6%)
12. Worried about being treated differently *	395 (37.4%)	199 (18.8%)	257 (24.3%)	143 (13.5%)	63 (6%)
13. Found health/other services difficult to cope with *	328 (31%)	206 (19%)	296 (28%)	151 (14.3%)	76 (7.2%)
14. Found treatments difficult to cope with *	361 (34.2%)	234 (22.1%)	280 (26.5%)	110 (10.4%)	72 (6.8%)
15. Felt that your health conditions made you unhappy *	249 (23.6%)	182 (17.2%)	378 (35.8%)	181 (17.1%)	67 (6.3%)
16. Felt you knew enough about your health conditions	101 (9.6%)	119 (11.3%)	296 (28%)	339 (32.1%)	202 (19.1%)
17. Had enough social contact with people	55 (5.2%)	103 (9.7%)	256 (24.2%)	350 (33.1%)	293 (27.7%)
18. Had enough support to cope well with health conditions	84 (7.9%)	141 (13.3%)	280 (26.5%)	306 (28.9%)	246 (23.3%)
19. Felt confident in managing health conditions	65 (6.1%)	122 (11.5%)	258 (24.4%)	371 (35.1%)	241 (22.8%)
20. Able to live your life as you want	83 (7.9%)	119 (11.3%)	307 (29%)	340 (32.2%)	208 (19.7%)

* Reverse-coded item.

**Table 3:** Items outfits, infits, locations, Thurstonian thresholds, and DIF.

Items	EFA Loadings (F1)	EFA Loadings (F2)	Communality	CFA Loading	Outfit	Infit	Location	Thresholds				DIF
								1	2	3	4	
LTCQ 1	0.55	0.09	0.35	0.62	1.16	1.12	−0.32	−1.47	−0.99	−0.03	1.31	0.00
LTCQ 2	0.77	0.0	0.59	0.75	0.84	0.87	−0.75	−1.77	−1.18	−0.45	0.83	0.00
LTCQ 3	0.7	−0.02	0.48	0.71	0.94	0.96	−0.44	−1.90	−1.11	0.04	1.42	−0.01
LTCQ 4	0.72	−0.02	0.51	0.73	0.89	0.94	−0.64	−1.84	−1.18	−0.33	1.17	0.05
LTCQ 5	0.7	−0.04	0.47	0.66	1.00	1.02	−0.47	−1.87	−1.04	−0.08	1.34	−0.02
LTCQ 6	0.7	−0.02	0.48	0.58	1.07	1.05	−0.74	−1.64	−1.06	−0.46	0.60	−0.01
LTCQ 7	0.71	0.05	0.53	0.68	0.85	0.95	−1.19	−1.86	−1.25	−0.75	−0.10	0.03
LTCQ 8	0.66	−0.07	0.41	0.5	1.23	1.17	−0.33	−1.56	−0.88	0.00	1.24	0.11
LTCQ 10	−0.06	0.62	0.37	0.68	1.11	1.11	−0.74	−1.95	−1.03	−0.26	0.72	−0.03
LTCQ 11	0.06	0.78	0.65	0.86	0.82	0.88	−0.80	−1.86	−0.93	−0.24	0.34	0.01
LTCQ 12	0.0	0.81	0.65	0.8	0.86	0.92	−0.84	−1.83	−0.97	−0.26	0.25	−0.04
LTCQ 13	−0.04	0.67	0.43	0.52	1.15	1.14	−0.67	−1.73	−0.91	−0.11	0.47	0.04
LTCQ 14	−0.02	0.7	0.48	0.67	1.00	1.03	−0.78	−1.66	−1.05	−0.27	0.38	−0.04
LTCQ 15	0.05	0.67	0.48	0.7	1.02	1.03	−0.53	−1.92	−0.91	0.19	0.77	−0.06
LTCQ 16	0.59	−0.19	0.31	0.48	1.48	1.31	−0.39	−1.46	−0.92	−0.13	1.12	−0.01
LTCQ 17	0.71	0.03	0.51	0.65	0.96	0.97	−0.78	−1.82	−1.16	−0.41	0.70	−0.03
LTCQ 18	0.67	0.03	0.46	0.68	1.00	1.00	−0.52	−1.63	−0.92	−0.16	0.87	0.02
LTCQ 19	0.67	0.06	0.47	0.72	0.94	0.97	−0.63	−1.76	−1.05	−0.32	0.95	0.01
LTCQ 20	0.73	0.08	0.59	0.75	0.87	0.90	−0.47	−1.60	−1.00	−0.15	1.09	−0.01

F1—Empowerment and Functional Wellbeing factor, F2—Health-Related Psychosocial Distress factor.

## Data Availability

The dataset supporting the findings of this article is available in [Zenodo] at [https://doi.org/10.5281/zenodo.15303249].

## Abbreviations

The following abbreviations are used in this manuscript:

CFA	Confirmatory factor analyses
CFI	Comparative Fit Index
DIF	Differential item functioning
EFA	Exploratory factor analyses
EQ-5D	EuroQol 5 Dimensions
KMO	Kaiser–Meyer–Olkin
LTCQ	Long-Term Conditions Questionnaire
MinRes	Minimum residual
MSE	Outfit mean square
NCCDs	Non-communicable chronic diseases
PROMs	Patient-reported outcome measures
RMSEA	Root Mean Square Error of Approximation
SRMR	Standardized Root Mean Square Residual
TLI	Tucker–Lewis Index
WLSMV	Weighted Least Squares Mean and Variance adjusted
